# Retracted: Flavonoid-Deficient Mutants in Grass Pea (*Lathyrus sativus* L.): Genetic Control, Linkage Relationships, and Mapping with Aconitase and S-Nitrosoglutathione Reductase Isozyme Loci

**DOI:** 10.1155/2016/2584053

**Published:** 2016-09-01

**Authors:** The Scientific World Journal

The Scientific World Journal has retracted the article titled “Flavonoid-Deficient Mutants in Grass Pea (*Lathyrus sativus* L.): Genetic Control, Linkage Relationships, and Mapping with Aconitase and S-Nitrosoglutathione Reductase Isozyme Loci” [1]. The article was found to contain images with signs of duplication and manipulation in Figures 2 and 3.
